# Assessment of remote ischemic conditioning delivery with optical sensor in acute ischemic stroke: Randomised clinical trial protocol

**DOI:** 10.1371/journal.pone.0284879

**Published:** 2023-05-04

**Authors:** Radhika Nair, Robert Sarmiento, Asif Sheriff, Ashfaq Shuaib, Brian Buck, Michel Gauthier, Vivian Mushahwar, Martin Ferguson-Pell, Mahesh Kate

**Affiliations:** 1 Department of Medicine, University of Alberta, Edmonton, Alberta, Canada; 2 Department of Rehabilitation Medicine, University of Alberta, Edmonton, Alberta, Canada; Public Library of Science, UNITED STATES

## Abstract

**Background:**

Remote ischemic conditioning (RIC) is delivered by a blood pressure cuff over the limb, raising pressure 50 mmHg above the systolic blood pressure, to a maximum of 200 mmHg. The cuff is inflated for five minutes and then deflated for five minutes in a sequential ischemia-reperfusion cycle 4–5 times per session. Elevated pressure in the limb may be associated with discomfort and consequently reduced compliance. Continuous assessment of relative blood concentration and oxygenation with a tissue reflectance spectroscopy (a type of optical sensor device) placed over the forearm during the RIC sessions of the arm will allow us to observe the effect of inflation and deflation of the pressure cuff. We hypothesize, in patients with acute ischemic stroke (AIS) and small vessel disease, RIC delivered together with a tissue reflectance sensor will be feasible.

**Methods:**

The study is a prospective, single-center, randomized control trial testing the feasibility of the device. Patients with AIS within 7 days from symptoms onset; who also have small vessel disease will be randomized 2:1 to intervention or sham control arms. All patients randomized to the intervention arm will receive 5 cycles of ischemia/reperfusion in the non-paralyzed upper limb with a tissue reflectance sensor and patients in the sham control arm will receive pressure by keeping the cuff pressure at 30 mmHg for 5 minutes. A total of 51 patients will be randomized, 17 in the sham control arm and 34 in the intervention arm. The primary outcome measure will be the feasibility of RIC delivered for 7 days or at the time of discharge. The secondary device-related outcome measures are fidelity of RIC delivery and the completion rate of intervention. The secondary clinical outcome includes a modified Rankin scale, recurrent stroke and cognitive assessment at 90 days.

**Discussion:**

RIC delivery together with a tissue reflectance sensor will allow insight into the blood concentration and blood oxygenation changes in the skin. This will allow individualized delivery of the RIC and improve compliance.

**Clinical trial registration:**

ClinicalTrials.gov Identifier: NCT05408130, June 7, 2022.

## Background and rationale

Cerebral small vessel disease (cSVD) is common and characterized by the presence of white matter lesions, microbleeds, prominent perivascular spaces, lacunar infarcts and intracerebral hemorrhages. The presence of cSVD is associated with an increased risk of stroke recurrence, worse functional outcome and persistent cognitive impairment following an acute ischemic stroke [1]. Current standard of care focuses on control of vascular risk factors including hypertension, diabetes, dyslipidemia, smoking, and physical activity to reduce the impact of cSVD on stroke outcomes. Despite the disease burden, there is no targeted therapy available for reducing the burden of SVD available currently. In animal models for vascular contributions to cognitive impairment and dementia, RIC for one month demonstrated improved cerebral blood flow, prevented white matter damage, increased angiogenesis and improved cognitive outcomes [2]. In a pilot human study, RIC for one year led to a reduction in white matter hyperintensities volumes [3].

Remote Ischemic Conditioning (RIC) is the technique of transient focal ischemia and reperfusion to a vascular bed, tissue, or organ. This may increase tissue tolerance to ischemia and prevent damage due to the ensuing cascade [4]. In a canine model, Murry et al. found that occlusion of the coronary circumflex artery for 5 minutes followed by reperfusion for 5 mins, in a cyclical manner 4 times before inducing experimental myocardial infarction in the same arterial territory resulted in 75% reduction in the infarct size compared to controls [5]. Subsequent experiments observed that effect of transient cyclical ischemia-reperfusion is transferable in different coronary artery territory also induced ischemia tolerance when compared to the control in varying degrees [6]. Further studies showed transient cyclical ischemia-reperfusion to a limb muscles or in a remote organ can offer protection not only against heart, but also other organs including brain, liver, and kidneys [7–10]. The mechanism of action of remote ischemic conditioning is through humoral, neural and immunomodulatory pathways [11]. Early-phase 2 and a few phase 3 human clinical trials on RIC in ischemic stroke have been promising so far [12–15].

Current RIC standard practice is to deliver ischemia by increasing the pressure in the arm BP cuff by 50 mmHg above the systolic blood pressure (to a maximum of 200 mmHg). These high pressures may be associated with discomfort, consequently reducing the patient’s compliance with the intervention [12]. The required pressure to deliver ischemia may be variable in different subjects. It will be important to deliver pressure comfortably to the patient. A skin tissue reflectance spectroscopy (a type of optical sensor) may allow assessment of the blood concentration and blood oxygenation during the delivery of RIC. This may allow the identification of precise RIC pressure delivery for each patient.

Our group has developed a skin tissue reflectance spectroscopy device for RIC delivery monitoring. In this clinical trial, we will assess the device’s feasibility. We hypothesize, in patients with ischemic stroke and small vessel disease, RIC delivered together with a tissue reflectance sensor will be feasible.

## Methods

### Device development

A prolonged occlusion arm pressure cuff was developed using an off-the-shelf Blood Pressure(BP) monitor, and a solenoid-actuated valve was added that allows pressure feedback to sustained pressure for 5 min. This added component enabled the blood pressure machine provide two modes:BP mode which assesses the systolic/diastolic BP and Ischemic; conditioning mode which sustains pressure for 5 min followed by deflation for 5 cycles. The optical sensor comprised a 3-wavelength high output LED (Marktech MTMD6894T38) operating at 670, 810, 950nm, a regulated LED driver circuit and a microcontroller (Adafruit M0 datalogger) that controlled the sequence for illuminating the LEDs and processed the relative amplitude of the backscattered light from the skin that was detected with an array of OPT101 (Text Instruments) photodetectors.

The LED is placed in the centre and is surrounded by the photodetectors. The sensor estimated the relative blood concentration and oxygenation in real-time during RIC therapy inflation and deflation. It wirelessly transmitted the measurements to a laptop computer that stored the readings and provided the clinician with a dashboard to monitor the readings throughout the treatment cycle. The indices of blood oxygenation (2 measures) and concentration were calculated using the following formulas:

$$\text{Blood}\;\text{Concentration}={\text{log}}_{10}(\left(\text{Signal}\;\text{at}\;816\text{nm}\right)/\left(\text{Resting}\;\text{level}\;\text{of}\;816\text{nm}\right))$$

$${\text{Blood}\;\text{Oxygenation}}_{768}=-1*\left({\text{log}}_{10}\left(\text{Signal}\;\text{at}\;768\text{nm}-\text{Signal}\;\text{at}\;816\text{nm}\right)/\left(\text{Resting}\;\text{level}\;\text{of}\;768\text{nm}\right)\right)$$

$${\text{Blood}\;\text{Oxygenation}}_{868}=-1*\left({\text{log}}_{10}\left(\text{Signal}\;\text{at}\;868\text{nm}-\text{Signal}\;\text{at}\;816\text{nm}\right)/\left(\text{Resting}\;\text{level}\;\text{of}\;868\text{nm}\right)\right)$$

By sampling the LEDs quickly and sampling at over 5 min complete measurements every second it is also possible to monitor the pulse rate as the blood concentration increases up to systole and decreases to diastole. There is a characteristic decay in the blood oxygenation during ischemia and then a rapid recovery with a small overshoot above baseline, and then a gradual return to baseline. These parameters can be used to characterize the difference in individual patients’ tissue responses.

### Study design and setting

**Fig 1:**
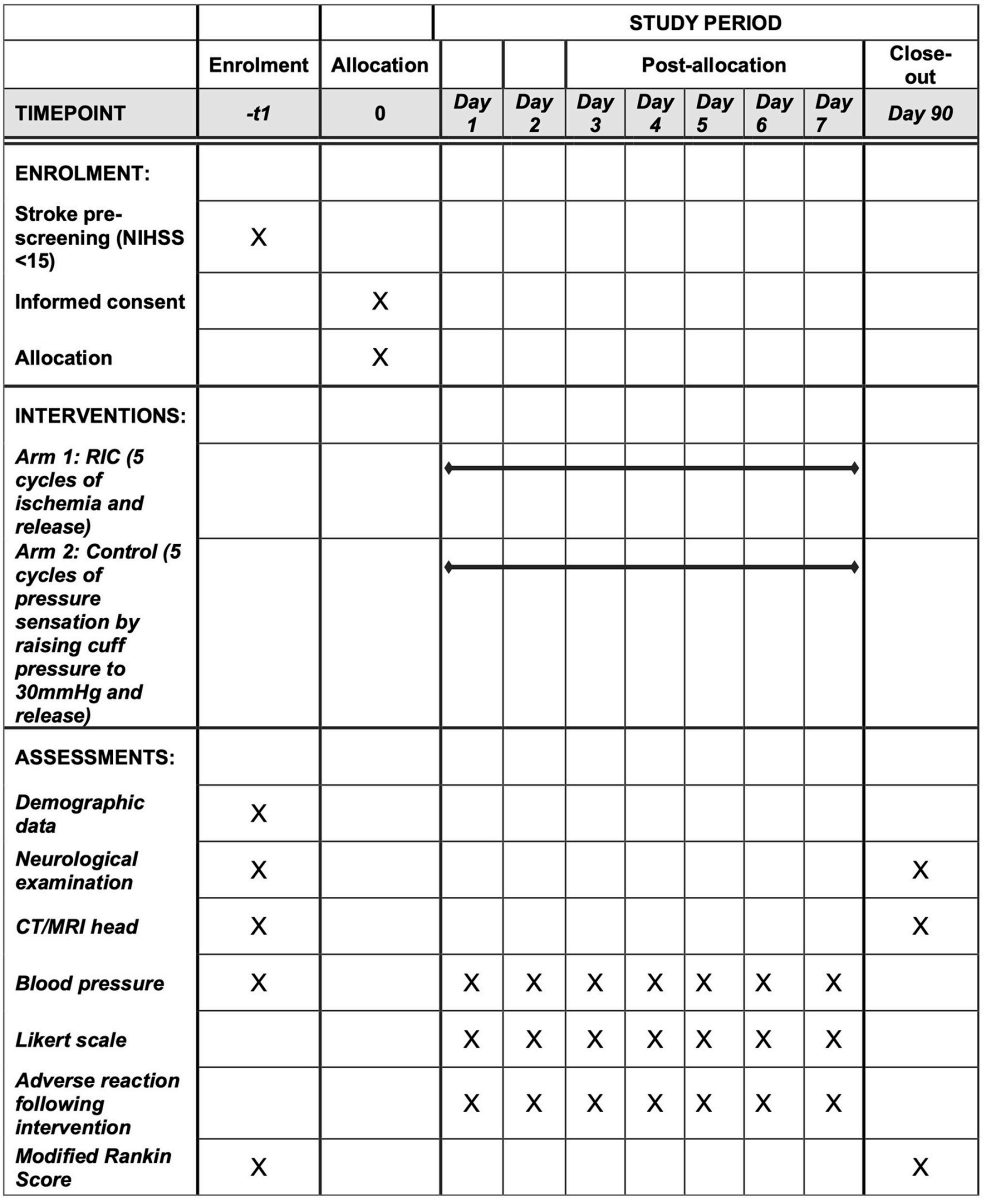
Study schedule.

The study is designed as a randomized control, single-blinded (patient), single-center, parallel-group, 1:2 randomization to sham control arm vs intervention arm, phase 2 clinical trial to assess the feasibility (Fig 1). The study has received ethics approval from the institutional ethics committee Human Research Ethics Board, University of Alberta (Pro00117448, April 8, 2022). Written informed consent will be obtained either from the patient or the substitute decision maker. The consent will be obtained by the person delivering therapy and monitored by another study personnel.

#### Eligibility criteria

Acute stroke patients within 48 hours from symptom onset will be screened. Adult patients aged 18 years or more with ischemic stroke (anterior and posterior circulation involvement) with or without neurological deficit, with evidence of infarct and small vessel disease on either computed tomography (CT) of the head or magnetic resonance imaging (MRI) of the brain, will be included after written informed consent is obtained from the patient or substitute decision maker. Infarct will be defined as new hypodensity in the CT head or hyperintensity in diffusion-weighted imaging with corresponding hypo-intensity in the apparent diffusion coefficient imaging. Small vessel disease will be defined as the presence of periventricular hyperintensities/hypodensities, deep white matter hyperintensities/hypodensities, microbleeds on MRI, new or old lacunar infarcts and Virchow Robin spaces in either CT or MRI. Patients with a pre-stroke functional disability as assessed by a modified Rankin scale of 2 or more, and a severe neurological deficit as assessed by the National Institute of Health Stroke Scale of 15 or more will be excluded. Patients receiving oral or parenteral anticoagulation therapy will be excluded. Patients with injury to the arm/forearm or any other musculoskeletal disability/pain and dermatological conditions affecting the application of tissue perfusion sensor and RIC pressure cuff will be excluded. Patients who are receiving ongoing treatment for active malignancy with expected survival <6 months, who have hypertensive urgency and emergency, ongoing systemic infection and hemodynamically instability will be excluded. Pregnant and lactating women will be excluded as the effect of RIC on the fetus is not known.

#### Interventions

**Fig 2:**
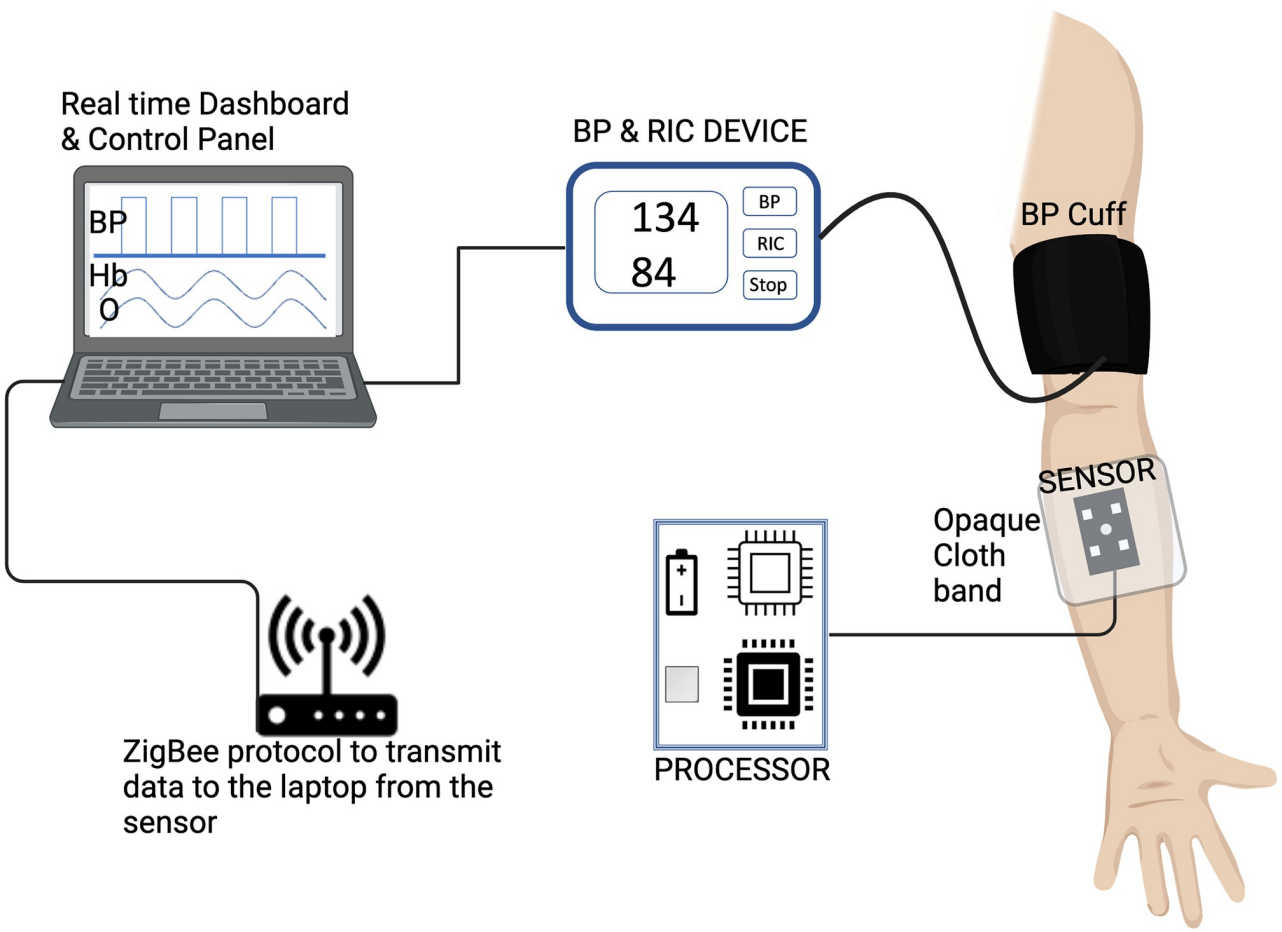
The setup for the remote ischemic conditioning delivery in the arm muscles. The tissue reflectance sensor is placed over the forearm and covered with an opaque cloth band for better-quality data acquisition and holding the sensor in place. The processor transmits the data in real time with a ZigBee protocol, with a mini-tower connected to the laptop for power. The dashboard synchronizes the data from the BP device and tissue reflectance sensor to display in real time.

All patients randomized to the intervention arm will receive 5 cycles of ischemia/reperfusion in the non-paralyzed upper limb with the use of an optical feedback sensor to keep the blood pressure target 30–50 mmHg above the systolic BP to a maximum of 200 mmHg. Each cycle will last for 5 minutes of ischemia (by inflating the blood pressure cuff above systolic BP) followed by 5 minutes of reperfusion (deflation of the cuff). If there is no upper limb paralysis, the non-dominant arm will be used. Patients will receive RIC therapy once daily for a period of 7 days or during their hospital stay whichever is shorter. The subjects in the sham group will receive a similar type and duration of pressure sensation by keeping the pressure at 30 mmHg in the non-paralyzed arm or if no upper limb paralysis non-dominant arm. All RIC therapy and sham control sessions will be monitored by the study personnel. The therapy sessions will be delivered at the bedside in the stroke unit (Fig 2).

#### Modification

During the RIC therapy, if the patient develops pain and wants to stop the procedure the session will be terminated immediately and documented as protocol deviation. The patient would be approached again the next day for the therapy session if they agree the therapy will be continued. However, if they disagree or the pain re-occurs it will be considered as termination of the trial. In situations of worsening of patient’s clinical conditioning which require the patient to be moved to a high care setting the RIC therapy will be temporarily stopped. The RIC therapy session will resume once the patient is clinically stable.

#### Concomitant care

All patients in the sham and intervention arm will receive standard of care management for ischemic stroke as per the Canadian Stroke Best Practices Guidelines [16].

#### Outcome

The primary outcome measure is the safety and feasibility of RIC delivered during the first 7 days or till discharge. Safety outcome measures will include assessing the level of comfort with the Likert scale after each therapy session. The Likert scale is a psychometric scale used for outcome assessment. The Likert scale will be used to measure comfort post-procedure and will be divided into 5 levels very comfortable, comfortable, neither comfortable nor uncomfortable, uncomfortable and very uncomfortable [17]. Persistent safety concerns lasting for >10 minutes after completion of RIC will also be recorded, that included assessment for pain or any persistent bruises. Other RIC therapy-related outcome measures include completion rate of intervention. The secondary clinical outcome includes mRS assessed at 90 days via telephone by a blinded observer, recurrent stroke and cognitive assessment at 90 days.

#### Assignment and concealment of intervention

A 1:2 block randomization list was generated using sealedenvelope.com and kept with a blinded study investigator. Participants will be randomly assigned to either a sham or intervention group according to the list. The study investigator enrolling the patient will call the blinded study investigator to get a randomization group.

#### Blinding

The patient will be blinded to the therapy arm. Assessment regarding comfort using the Likert scale and persistent safety concerns lasting for >10 minutes will be assessed by the study investigator delivering the RIC therapy session. The mRS, cognitive assessment and recurrent stroke at 90 days will be assessed by a blinded investigator to the therapy arm. Due to the nature of the intervention, the investigator delivering therapy cannot be blinded to allocation.

#### Data collection

Patient demographic, clinical, laboratory, imaging and medication details will be abstracted on an electronic case record form. Case record form (CRF) at baseline will include premorbid functional status, risk factor profile, vital signs, the arterial territory of qualifying stroke, revascularization therapy and imaging findings including the severity of small vessel diseases. We will assess BP prior to each intervention session. Patient comfort, measured by a Likert scale will be recorded after each cycle. Persistent safety concerns lasting for >10 minutes after completion of RIC will also be recorded. Follow-up in 3 months will be done through a telephone call.

#### Statistical method

**Fig 3:**
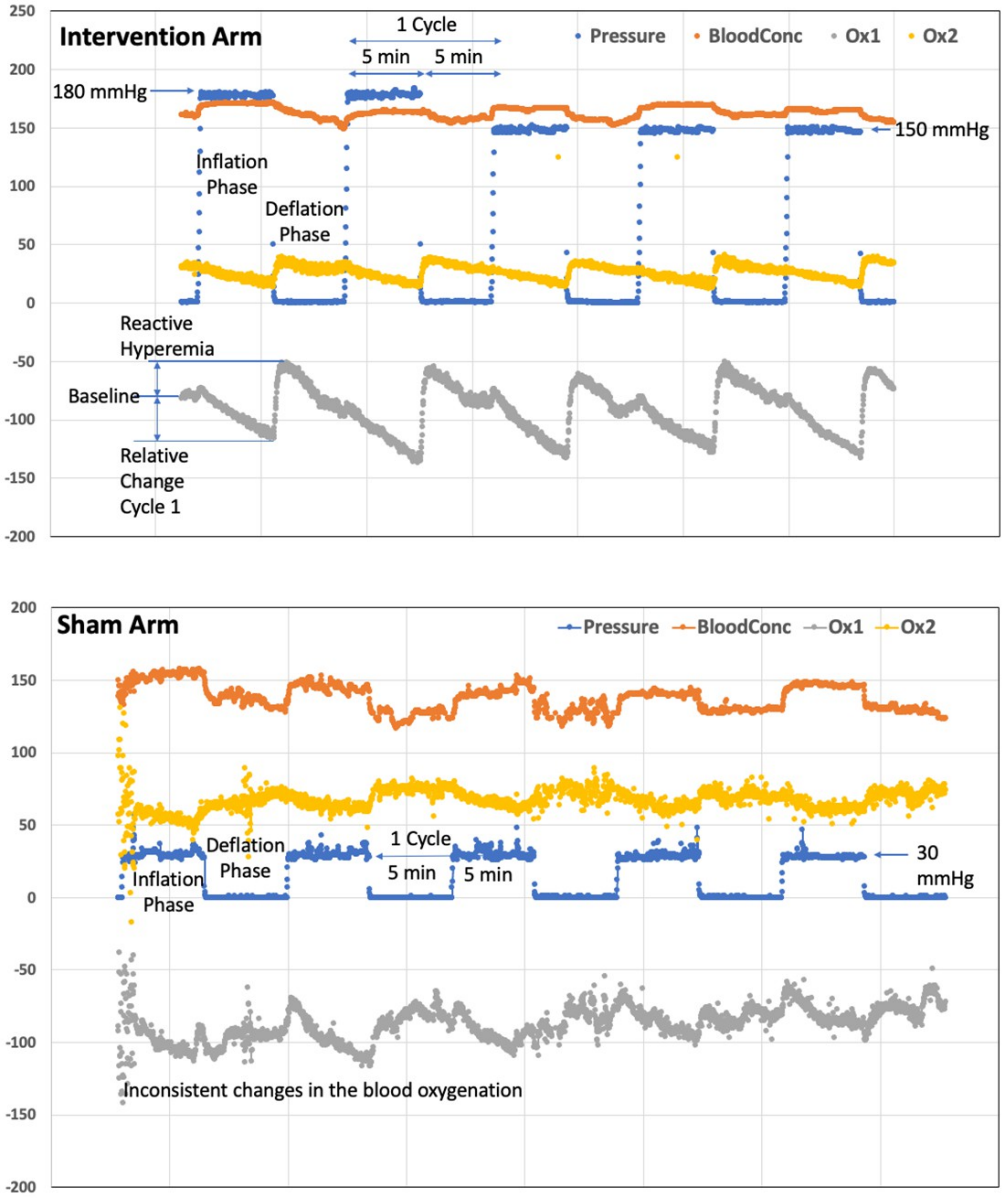
Systolic blood pressure (SBP), blood concentration (BloodConc), and blood oxygenation changes (Ox1 and Ox 2) during a remote ischemic conditioning therapy session with 5 cycles in the intervention arm and sham arm. *Intervention Arm*: The SBP (blue) was maintained at 180 mmHg for the initial two cycles and 150 mmHg for the remaining three cycles. The BloodConc (orange) increases with inflation and decreases with deflation. Ox1 and Ox2 (gray and yellow) decrease with inflation and bounces back with deflation. There is transient reactive hyperemia (increase in Ox1 and Ox2) corresponding to the early phase of deflation. There was no apparent change noted in the BloodConc, Ox1 and Ox2 after the change in the blood pressure from 180 mmHg to 150 mmHg. *Sham Arm*: the SBP was maintained at 30 mmHg for the five cycles. The BloodConc, Ox1 and Ox2 change inconsistently.

We will enroll a total of 51 (17 in Sham Control and 34 in the Intervention arm) patients over a 9-month period. The stroke risk factors will be reported as percentages. NIHSS will be reported as median with interquartile range. The Likert scale will be used to measure the comfort post-procedure, the Likert scale is 5 points ordinal scale to which the patient agrees or disagree with the procedure. Each group will be divided into two groups who achieved good feasibility Likert scale <3 and not feasible Likert scale >2. They will be compared with the Chi-Square test or Fischer Exact test. The safety endpoint and completion rate will be described as percentages. The 1:2 randomization will allow better detection of feasibility and safety concerns. The median (IQR) blood concentration and median (IQR) oxygen concentration (both frequencies 768 and 868) change for each cycle will be assessed for all sessions (Fig 3). The median change will be compared between the sham and intervention arm with a Mann-Whitney U test. The median change between individual sessions for the same patient will be assessed (IBM SPSS version 28 and significance were set at p<0.05).

## Summary

RIC-device therapy is promising, non-invasive and may be cost-effective in acute ischemic stroke. However real-time monitoring may allow insights into the delivery of RIC. A novel tissue reflectance sensor coupled with RIC therapy is being tested in this clinical trial. The changes in blood concentration and blood oxygenation during the delivery of RIC therapy will provide data on the physiological changes occurring in the skin capillaries. This will allow individualized delivery of the RIC and improve compliance.

## Supporting information

S1 Checklist SPIRIT 2013 checklist. (DOC)

S1 File Study protocol. (PDF)

## References

[1] Georgakis MK, Duering M, Wardlaw JM, Dichgans M. WMH and long-term outcomes in ischemic stroke: A systematic review and meta-analysis. Neurology. 2019;92:E1298–E1308. doi: 10.1212/WNL.0000000000007142 . 30770431

[2] Khan MB, Hafez S, Hoda MN, Baban B, Wagner J, Awad ME, et al. Chronic Remote Ischemic Conditioning Is Cerebroprotective and Induces Vascular Remodeling in a VCID Model. Transl Stroke Res. Translational Stroke Research; 2018;9:51–63. doi: 10.1007/s12975-017-0555-1 28755277 PMC5750336

[3] Wang Y, Meng R, Song H, Liu G, Hua Y, Cui D, et al. Remote ischemic conditioning may improve outcomes of patients with cerebral small-vessel disease. Stroke. 2017;48:3064–3072. doi: 10.1161/STROKEAHA.117.017691 . 29042490

[4] Heusch G, Bøtker HE, Przyklenk K, Redington A, Yellon D. Remote ischemic conditioning. J Am Coll Cardiol. 2015 Jan 20;65(2):177–95. doi: 10.1016/j.jacc.2014.10.031 . 25593060 PMC4297315

[5] Murry CE, Jennings RB, Reimer KA. Preconditioning with ischemia: a delay of lethal cell injury in ischemic myocardium. Circulation. 1986 Nov;74(5):1124–36. doi: 10.1161/01.cir.74.5.1124 . 3769170

[6] Przyklenk K, Bauer B, Ovize M, Kloner RA, Whittaker P. Regional ischemic ’preconditioning’ protects remote virgin myocardium from subsequent sustained coronary occlusion. Circulation. 1993 Mar;87(3):893–9. doi: 10.1161/01.cir.87.3.893 . 7680290

[7] Baig S, Moyle B, Nair KPS, Redgrave J, Majid A, Ali A. Remote ischaemic conditioning for stroke: unanswered questions and future directions. Stroke Vasc Neurol. 2021 Jun;6(2):298–309. doi: 10.1136/svn-2020-000722 . 33903181 PMC8258051

[8] Candilio L, Malik A, Hausenloy DJ. Protection of organs other than the heart by remote ischemic conditioning. J Cardiovasc Med (Hagerstown). 2013 Mar;14(3):193–205. doi: 10.2459/JCM.0b013e328359dd7b . 23079610

[9] Wever KE, Warlé MC, Wagener FA, van der Hoorn JW, Masereeuw R, van der Vliet JA, et al. Remote ischaemic preconditioning by brief hind limb ischaemia protects against renal ischaemia-reperfusion injury: the role of adenosine. Nephrol Dial Transplant. 2011 Oct;26(10):3108–17. doi: 10.1093/ndt/gfr103 . 21427077

[10] Tapuria N, Kumar Y, Habib MM, Abu Amara M, Seifalian AM, Davidson BR. Remote ischemic preconditioning: a novel protective method from ischemia reperfusion injury—a review. J Surg Res. 2008 Dec;150(2):304–30. doi: 10.1016/j.jss.2007.12.747 . 19040966

[11] Abbasi-Habashi S, Jickling GC, Winship IR. Immune Modulation as a Key Mechanism for the Protective Effects of Remote Ischemic Conditioning After Stroke. Front Neurol. 2021; 12:746486. doi: 10.3389/fneur.2021.746486 . 34956045 PMC8695500

[12] Kate M, Brar S, George U, Rathore S, Butcher K, Pandian J, et al. Self- or caregiver-delivered manual remote ischemic conditioning therapy in acute ischemic stroke is feasible: the Early Remote Ischemic Conditioning in Stroke (ERICS) trial. Wellcome Open Res. 2019;4:147.

[13] An JQ, Cheng YW, Guo YC, Wei M, Gong MJ, Tang YL, et al. Safety and efficacy of remote ischemic postconditioning after thrombolysis in patients with stroke. Neurology. 2020;95:e3355–e3363. doi: 10.1212/WNL.0000000000010884 . 33028663

[14] Meng R, Asmaro K, Meng L, Liu Y, Ma C, Xi C et al. Upper limb ischemic preconditioning prevents recurrent stroke in intracranial arterial stenosis. Neurology. 2012;79:1853–1861. doi: 10.1212/WNL.0b013e318271f76a 23035060

[15] Chen HS, Cui Y, Li XQ, Wang XH, Ma YT, Zhao Y, et al. Effect of Remote Ischemic Conditioning vs Usual Care on Neurologic Function in Patients With Acute Moderate Ischemic Stroke: The RICAMIS Randomized Clinical Trial. *JAMA*. 2022;328(7):627–636. doi: 10.1001/jama.2022.13123 . 35972485 PMC9382441

[16] Heran M, Lindsay P, Gubitz G, Yu A, Ganesh A, Lund R, et al. Canadian Stroke Best Practice Recommendations: Acute Stroke Management, 7^th^ Edition Practice Guidelines Update, 2022. *Can J Neurol Sci*. 2022;1–94. 10.1017/cjn.2022.344 36529857

[17] Sullivan GM, Artino AR Jr. Analyzing and interpreting data from Likert-type scales. J Grad Med Educ. 2013 Dec;5(4):541–2. doi: 10.4300/JGME-5-4-18 24454995 PMC3886444

