# Supplementary material for: Assessment of remote ischemic conditioning delivery with optical sensor in acute ischemic stroke: Randomised clinical trial protocol
Source: PLoS One. 2023 May 4;18(5):e0284879. doi: 10.1371/journal.pone.0284879 (PMC10159200; doi:10.1371/journal.pone.0284879)
Supplement: S1 File — (PDF) [file pone.0284879.s002.pdf]

Date: Monday, March 20, 2023 5:38:13 PM

Print Close

Table of Contents

Pro00117448

Packet Name: 2 - Smartform

1.1 Study Identification
1.2 Additional Approval
1.3 Funding Information
1.4 Conflict of Interest
1.5 Research Locations and Other Approvals
2.1 Study Objectives and Design
2.2 Research Methods and Procedures
2.16 Clinical Trial
2.17 Data Safety and Monitoring for Clinical Trials
2.19 Investigational Drugs, Devices, Biologics, Vaccines or Natural Health Products
3.1 Risk Assessment
3.2 Benefits Analysis
4.1 Participant Information
4.2 Additional Participant Information
4.3 Recruitment of Participants (Health)
4.5 Informed Consent Determination
5.1 Data Collection
5.2 Data Identifiers
5.3 Data Confidentiality and Privacy
5.4 Data Storage, Retention, and Disposal
Documentation
Final Page
Add/Edit Funding Info without Manual Entry (ID00064677)

Packet Name: 3 - Reviewer Notes

Reviewer Notes

1.1 Study Identification

*All questions marked by a red asterisk * are required fields. However, because the mandatory fields have been kept to a minimum, answering only the required fields may not be sufficient for the REB to review your application.*

Please answer all relevant questions that will reasonably help to describe your study or proposed research.

1.0 * **Short Study Title** *(restricted to 250 characters)*:
Remote Ischemic Conditioning with Novel Optical Sensor Feedback Device in Acute Ischemic Stroke

2.0 * **Complete Study Title** *(can be exactly the same as short title)*:
Remote Ischemic Conditioning with Novel Optical Sensor Feedback Device in Acute Ischemic Stroke

3.0 * **Select the appropriate Research Ethics Board (***Detailed descriptions are available at here***):**
HREB Biomedical

4.0 * **Is the proposed research:**
Funded (Grant, subgrant, contract, internal funds, donation or some other source of funding)

5.0 * **Name of local Principal Investigator:**
Mahesh Kate

6.0 * **Type of research/study:**
Faculty/Academic Staff

7.0 **Investigator's Supervisor***(required for applications from undergraduate students, graduate students, post-doctoral fellows and medical residents to REBs 1 & 2. HREB does not accept applications from student PIs)*:

8.0 **Study Coordinators or Research Assistants:** People listed here can edit this application and will receive all email notifications for the study:

Name	Employer
There are no items to display	

9.0 **Co-Investigators:** People listed here can edit this application and will receive email notifications *(Co-investigators who do not wish to receive email, should be added to the study team below instead of here).*
If your searched name does not come up when you type it in the box, the user does not have the Principal Investigator role in the online system. Click the following link for instructions on how to Request an Additional Role.

Name	Employer
Robert Sarmiento	Alberta Health Services (Department)
Radhika Kadangot	MH Medicine
Martin Ferguson-Pell	RM Dean Rehab Medicine

10.0 **Primary Admin Contact** *(a member of study team)*:

11.0 **Study Team:** *(co-investigators, supervising team, and other study team members) - People listed here cannot view or edit this application and do not receive email notifications.*

Last Name	First Name	Organization	Role/Area of Responsibility	Phone	Email
Mushahwar	Vivian	University of Alberta	Co-Investigator		
Buck	Brian	University of Alberta	Co-Investigator		
Michelle	Whaling	University of Alberta			whaling@ualberta.ca
Michel	Gauthier	University of Alberta	Research Associate		mgauthie@ualberta.ca

ID: Pro00117448
Status: Approved

Pro00117448

1.2 Additional Approval

1.2 Additional Approval

1.0 * **Departmental Review:** ***Please note only ONE Department Review is required. Please ensure that this section reflects only the PRIMARY Department of the study PI.***

MH Medicine

2.0 **Internal Review** *(If the Principal Investigator is in the Department of Medicine complete the Department of Medicine Request for Internal Approval form and upload it to the "Documentation" section of this application under item 11.0 "Other Documents". Note that all fields in the form are required. The form is available at here)*:

Medicine

ID: Pro00117448
Status: Approved

Pro00117448

1.3 Funding Information

1.3 Study Funding Information

1.0 * **Type of Funding:**
Grant (external)

2.0 * **Indicate which office administers your award.** *(It is the PI's responsibility to provide ethics approval notification to any office other than the ones listed below)*
University of Alberta - Research Services Office (RSO)

To connect your ethics application with your funding: provide all identifying information about the study funding – multiple rows allowed. For Project ID, enter a Funding ID provided by RSO/PeopleSoft Project ID*(for example, RES0005638, G018903401, C19900137, etc).***Enter the corresponding title for each Project ID.**

	Project ID	Title	Grant Status	Sponsor	Project Start Date	Project End Date	Purpose	Other Information
View	RES0055934	Remote Ischemic Conditioning with Novel Optical Sensor Feedback Device in Acute Ischemic Stroke	Awarded		1/1/2022	12/31/2023	Grant	

3.0 * **Funding Source**

3.1 Select all sources of funding from the list below:
University Hospital Foundation UHF

3.2 If your source of funding is not available in the list above, click "Add" below and write the Sponsor/Agency name(s) in the free text box that pops up.*(Nore: You may reflect multiple sources of funding by continuing to click "Add" to add each additional source of funding).*
There are no items to display

4.0 * **Indicate if this research sponsored or monitored by any of the following:**
Not applicable

The researcher is responsible for ensuring that the study complies with the applicable US regulations. The REB must also comply with US Regulations.

ID: Pro00117448 **Pro00117448** 1.4 Conflict of Interest
Status: Approved

1.4 Conflict of Interest

1.0 * **Are any of the investigators or their immediate family receiving any personal remuneration (including investigator payments and recruitment incentives but excluding trainee remuneration or graduate student stipends) from the funding of this study that is not accounted for in the study budget?**

○ Yes ● **No**

2.0 * **Do any of investigators or their immediate family have any proprietary interests in the product under study or the outcome of the research including patents, trademarks, copyrights, and licensing agreements?**

● **Yes** ○ No

3.0 * **Is there any compensation for this study that is affected by the study outcome?**

○ Yes ● **No**

4.0 * **Do any of the investigators or their immediate family have equity interest in the sponsoring company? (This does not include Mutual Funds)**

○ Yes ● **No**

5.0 * **Do any of the investigators or their immediate family receive payments of other sorts, from this sponsor (i.e. grants, compensation in the form of equipment or supplies, retainers for ongoing consultation and honoraria)?**

○ Yes ● **No**

6.0 * **Are any of the investigators or their immediate family, members of the sponsor's Board of Directors, Scientific Advisory Panel or comparable body?**

○ Yes ● **No**

7.0 * **Do you have any other relationship, financial or non-financial, that, if not disclosed, could be construed as a conflict of interest?**

○ Yes ● **No**

Please explain if the answer to any of the above questions is Yes:
The investigators have developed the tool, however no patents have been filed hence there is no financial gain.
To mitigate any conflict of interest we have taken the following steps:
1. The study intervention will be performed and documented by RK who is not part of the device development team
2. The study randomization will be performed by BB who is not part of the device development team

Important
If you answered YES to any of the questions above, you may be asked for more information.

ID: Pro00117448 **Pro00117448**
Status: Approved

1.5 Research Locations and Other Approvals

1.5 Research Locations and Other Approvals

1.0 * **List the locations of the proposed research, including recruitment activities. Provide name of institution, facility or organization, town, or province as applicable**
University of Alberta Hospital, Edmonton, Alberta

2.0 * **Indicate if the study will use or access facilities, programmes, resources, staff, students, specimens, patients or their records, at any of the sites affiliated with the following** *(select all that apply):*
Alberta Health Services Institutions and Facilities

List all health care research sites/locations:
University of Alberta Hospital (Stroke Unit and Stroke Wards)

3.0
Multi-Institution Review

* **3.1 Has this study already received approval from another REB?**
○ **Yes** ● **No**

4.0 **If this application is closely linked to research previously approved by one of the University of Alberta REBs or has already received ethics approval from an external ethics review board***(s)*, **provide the study number, REB name or other identifying information. Attach any external REB application and approval letter in the Documentation Section – Other Documents.**

ID: Pro00117448 **Pro00117448** 2.1 Study Objectives and Design
Status: Approved

2.1 Study Objectives and Design

1.0 * **Provide a lay summary of your proposed research which would be understandable to general public**

Stroke is a leading cause of physical and cognitive disabilities. The most common type of stroke is ischemic (lack of blood flow to the brain due to clot blocking a blood vessel). Many people with stroke (PwS) have changes on the brain imaging called small vessel disease (SVD). This is a condition that affects tiny blood vessels supplying the brain, leading to decreased blood flow in some parts of the brain. These brain changes may hamper the recovery process after stroke, or lead to recurrent stroke and cognitive impairment. SVD is a slow process that can be seen as multiple black spots on computed tomography or white spots on magnetic resonance imaging. Current treatments to reduce the effect of SVD on PwS are to control high blood pressure, high blood sugar, high cholesterol and increase physical activity. However, these approaches do not lead to a reduction in SVD.

Remote Ischemic Conditioning is a type of treatment delivered with help of a regular blood pressure machine. This does not involve any drug. A typical treatment involves the application of a blood pressure cuff followed by brief sessions of compressions and relaxation on the arm muscles much akin to blood pressure measurement but for 5 min. It leads to a transient safe state of less blood flow in arm muscles which initiates the

release of molecules and signals transmitted by blood. These signals may then go on to improve blood flow in the brain. Recent animal and human studies have suggested that the use of RIC may reduce the SVD load.

We have developed a new device to deliver remote ischemic conditioning therapy in a better manner. Existing devices generate the same amount of compression for all people. The pressure applied by the machine in the arm may be either more than required or less than required. The ideal compression would be one that achieves a low blood flow state in the arm at the least possible pressure. To achieve this our group is using a small light sensor to inform us. The light sensor is closely applied to the skin over the arm below the blood pressure cuff. It emits light that is absorbed by the skin and the light is then reflected. This is detected by other sensors placed together. From the reflected light the sensor can obtain information about blood flow in the skin. When the pressure increases with help of an automated machine the light sensor can detect that blood flow are reduced and this information is displayed on the computer. The information about skin blood flow will inform us about the level of pressure we have to apply to give accurate treatment.

We propose that our device with optical feedback will deliver RIC in PwS and SVD in a safe and reliable manner. A total of 51 participants will take part in this study. Thirty-four participants will get remote ischemic conditioning therapy and 17 patients will get sham-control therapy. All participants will get standard post-stroke treatment according to the Canadian Stroke Best Practices Recommendation.

2.0 * **Provide a full description of your research proposal outlining the following:**

- **Purpose**
- **Hypothesis**
- **Justification**
- **Objectives**
- **Research Method/Procedures**
- **Plan for Data Analysis**

1. Hypothesis

We hypothesize in patients with ischemic stroke and small vessel disease, Remote Ischemic Conditioning (RIC) delivered by novel device with optical feedback sensor will be safe and feasible.

2. Rationale

Cerebral small vessel disease (SVD) is highly prevalent in aging societies and also in patients with ischemic stroke.SVD is independently associated with cognitive impairment and incidence of stroke.SVD is characterised by presence of white matter lesions (commonly seen as hyperintensities on magnetic resonance imaging (MRI) or hypodensities on computed tomography, CT), microbleeds, prominent perivascular spaces, lacunar infarcts and intracerebral hemorrhages. Risk of worse outcomes in patients with ischemic stroke as assessed by modified Rankin scale (mRS) >2 is increased by odds ratio (OR) of 1.96 if there is presence of white-matter hyperintensities (WMH). Furthermore, WMH are also associated with increased risk of recurrent stroke (OR 1.93), cognitive impairment (OR 2.22) and all-cause mortality (OR1.82). Current standard of care focuses on control of vascular risk factors including hypertension, diabetes, dyslipidemia, smoking, physical activity and mental health concerns to reduce the impact of WMH on stroke outcomes. There is no targeted therapy available for reducing the burden of SVD.

RIC involves brief cyclic ischemia (5 minutes, inflation of BP cuff) and reperfusion (5 minutes, deflation of BP cuff) of a distant organ (arm muscles) to protect at-risk (cerebral tissue) organ tissue by increasing

ischemia tolerance. In experimental stroke RIC alone or in combination revascularization therapy may have additive effect, improve collateral circulation and reduce the infarct size.Human clinical trial in ischemic stroke have been promising so far. In animal models for vascular contributions to cognitive impairment and dementia (VCID) RIC for 1 month demonstrated improved cerebral blood flow, prevented white matter damage, improved angiogenesis and cognitive outcomes. In a pilot human study RIC for 1 year lead to reduction in WMH.

An important ongoing issue is the fidelity of RIC delivered to the patients. Typically for ischemia to develop in the limb muscles, current standard practice is to increase the pressure in the limb 30-50 mmHg above the systolic blood pressure to maximum of 200 mmHg. However, this may be variable in individual subjects. Pressure in upper limb may be associated with discomfort and consequent reduced compliance.An effective solution would be to identify ideal pressure target for each individual person. Our group is developing a novel RIC device, which will identify precise BP target with optical feedback sensor to deliver optimal ischemia with least possible pressure. This will improve patient experience and improve fidelity of RIC.

3. Device Development Progress

SMARTNETWORK engineer has developed prolonged occlusion pressure cuff with Life Source Premium blood Pressure monitor (Model UA-787EJCN), and Solenoid actuated valve which allows pressure feedback to sustained pressure for 5 min. The current proof of concept automated blood pressure works in two modes, BP mode where it assess the systolic/diastolic BP and Ischemic Conditioning mode where it can sustain pressure for 5 min followed by deflation (Figure 1). He has developed algorithm to deliver 5 cycles in row. The technical PI (MFP) has developed programmable optical sensor feedback with Tissue Reflectance Spectroscopy. A high output monochromatic light emitting diodes (LED) is used as light source. The Optodes (receiving sensors) analyze the spectrum of back scattered light. The sensor is able to monitor blood concentration and oxygenation during RIC therapy inflation and deflation. To calculate the blood oxygenation and concentration following formulas were used:

Blood Concentration = $\log_{10(}$(Signal at 816nm)/(Resting level of 816nm))

Blood Oxygenation$_{768}$ = -1*($\log_{10}$(Signal at 768nm -Signal at 816nm)/(Resting level of 768nm))

Blood Oxygenation$_{868}$ = -1*($\log_{10}$(Signal at 868nm -Signal at 816nm)/(Resting level of 868nm))

By sampling the LEDs quickly and sampling at over 5 min complete measurements every second it is also possible to monitor the pulse rate as the blood concentration increases up to systole and decreases to diastole. A proof of concept sensor with these capabilities has been developed and preliminary testing has been successfully conducted. As we can see in graph there is a characteristic decay in the blood oxygenation during ischemia and then a rapid recovery with an small overshoot above baseline, and then gradual return to baseline. These parameters can be used to characterize difference in individual patient’s tissue response. We are currently testing delivery of feedback to the blood pressure device from the optical sensor. We aim to complete the task it over next 2 months

4. Methods

4. A Device Assembly Phase (1 month)

The SMART network team including MFP, VM and MG with the research associate will help in assembly of the device. The device has three components first is blood pressure machine. The machine able to deliver 5 cycles of inflation and deflation (each lasting for 5 minutes). The second component is optical sensor as described above and third component is the feedback program running on a laptop.

Optical Sensor

The Optical sensor will be placed over the arm below the blood pressure cuff. The sensor will conform to the arm contour. The sensor has five components, a light emitter and four light sensors. The light emitter is placed in the centre and light sensors are placed surrounding the light emitter. They are in close proximity to the skin and covered by black plastic covering to avoid external light source penetration. It will be applied to the same side where the intervention will be delivered by the automated blood pressure machine. A common dashboard will display both the pressure generated by the automated blood pressure machine and the blood oxygenation level assessed by the optical sensor. This will be seen in real-time. The information will guide the study team to deliver appropriate pressure to the arm. A working model has been developed.

4. B Clinical Trial (11 months)

4.B.1 Study population

All patients with ischemic stroke admitted to the University of Alberta Hospital stroke ward will be screened.

Inclusion criteria

1. Adult patients with ischemic stroke (anterior and posterior circulation involvement) with or without neurological deficit within 7 days of symptom onset
2. CT head or MRI Brain evidence of infarct
3. CT head or MRI Brain evidence of moderate or severe small vessel disease. We will assess atrophy, leukoaraiosis and old vascular lesions.
4. Premorbid functional disability assessed by mRS <2
5. National institute of Health Stroke scale <15 at the time of enrollment

Exclusion criteria

1. Patient is part of other clinical trial delivering intervention
2. Injury to the upper arm or any other musculoskeletal disability/pain precluding from tolerating RIC therapy
3. Treatment of ongoing malignancy with expected survival < 6 months
4. Presence of hypertensive urgency and emergency
5. Presence of hemodynamic instability
6. Presence of ongoing systemic infection with antibiotic therapy
7. Pregnant and lactating women
8. History of dermatological conditions affecting application of tissue perfusion sensor and remote ischemic conditioning pressure cuff

4.B.2 Study design

Prospective randomised control trial with blinded end-point. 1:2 randomisation to sham or intervention arm. We will use sealed envelope randomization.

4.B.3 Study protocol

Intervention

All patients randomized to intervention arm will receive 5 cycles of ischemia/reperfusion in non-paralysed upper limb or if no upper limb paralysis non-dominant arm. They will receive it once daily for a period of 7 days or during hospital stay whichever is shorter. The Stroke Fellow will help in delivering the RIC therapy. Cleaning protocol for the device will be followed as per Alberta Health Services guidelines.

Sham

In the sham group subjects will receive pressure sensation by keeping the pressure at 30 mmHg for 3 min in both arms All patients in sham and intervention group will receive standard of care management for ischemic stroke.

4.B.4 Study procedures

Patient demographic clinical, laboratory and imaging details will be abstracted on an electronic case record form. We will assess BP prior to each intervention session. Follow up in 3 months will be via telephone.

4.B.5 Outcome

The primary outcome measure is safety and feasibility of RIC delivered during the first 7 days or at the time of discharge. In safety we will assess level of comfort with Likert scale. The Likert scale will be divided in 5 levels as very comfortable, comfortable, neither comfortable or uncomfortable, uncomfortable and very uncomfortable. In safety we would assess pain or any persistent bruises. Secondary outcome measures related to the intervention include fidelity of treatment and completion rate of intervention. The secondary clinical outcome include mRS assessed at 90 days via telephone by a blinded observer, recurrent stroke and cognitive assessment at 90 days.

4.B.6 Statistical analysis

We will enroll a total of 51 (17 in Sham Control and 34 in the Intervention arm) patients over 9 months period. We will have two groups patients with intervention and patients with sham treatment. The Likert scale is ordinal. Each group will be divided in to two groups who achieved good feasibility Likert scale <3 and not feasible Likert scale >2. They will be compared with Chi-Square test. The safety endpoint will be described as percentages.

4.B.7 Recruitment Strategy

University of Alberta Hospital stroke program admits approximately 1,200 patients every year. We have a group of 5 research coordinators who will help in recruitment.

3.0 **Describe procedures, treatment, or activities that are above or in addition to standard practices in this study area** *(eg. extra medical or health-related procedures, curriculum enhancements, extra follow-up, etc)*:

All patients will receive standard of care according to the Canadian Stroke Best Practices Guidelines. In addition they will receive the study intervention or sham treatment in form of remote ischemic conditioning as described in protocol for 7 days or upto discharge. The patients or caregivers will be called at the end of 3 months to assess clinical outcome.

4.0 **If the proposed research is above minimal risk and is not funded via a competitive peer review grant or industry-sponsored clinical trial, the REB will require evidence of scientific review. Provide information about the review process and its results if appropriate.**

This is study is reviewed and funded by University Hospital Foundation Medical Research Competition

5.0 **For clinical trials, describe any sub-studies associated with this Protocol.**
No sub studies are planned

2.2 Research Methods and Procedures

Some research methods prompt specific ethical issues. The methods listed below have additional questions associated with them in this application. If your research does not involve any of the methods listed below, ensure that your proposed research is adequately described in Section 2.1: Study Objectives and Design or attach documents in the Documentation Section if necessary.

1.0 * **This study will involve the following***(select all that apply)*
Clinical Trial
Drugs, Medical Devices, Biologics or Vaccines and/or Natural Health Products

NOTE 1: Select this ONLY if your application SOLELY involves a review of paper charts/electronic health records/administrative health data to answer the research question. If you are enrolling people into a study and need to collect data from their health records in addition to other interventions, then you SHOULD NOT select this box.

NOTE 2: Select this option if this research ONLY involves analysis of blood/tissue/specimens originally collected for another purpose but now being used to answer your research question. If you are enrolling people into the study to prospectively collect specimens to analyze you SHOULD NOT select this box.

2.16 Clinical Trial

1.0 **Protocol**

1.1 Protocol Number (if applicable):

1.2 Clinical trials must be registered before participant recruitment can begin. Provide registry and registration number, e.g. clinicaltrials.gov:

2.0 **Is this an investigator-initiated clinical trial?**

* **Is this study authored and initiated by a researcher from the University of Alberta, Alberta Health Services and/or Covenant Health?**

(●) **Yes** ( ) No

* **Is this study authored or sponsored by any outside entity including, but not limited to, a pharmaceutical company or clinical research organization?**

( ) Yes (●) **No**

3.0 ***Does the study involve any of the following?**

Answer	Description
(●) **Yes** ( ) No	A drug, device, biologics, vaccine or natural health product not marketed in Canada?
( ) Yes (●) **No**	A comparative bioavailability trial?
( ) Yes (●) **No**	Use of a marketed drug, device, biologics, vaccine, or natural health product outside the parameters of its officially “approved use” by Health Canada?

If you have answered yes to any of the questions above, a Health Canada Clinical Trial Application (CTA) may be required. The investigator MUST coordinate with the University of Alberta - Quality Management in Clinical Research for all Health Canada clinical trials, as the University will be the named Sponsor of the trial. Please contact lori.anderson@ualberta.ca for assistance.

4.0 **Trial Phase:**
Phase II clinical trials study the biomedical or behavioral intervention in a larger group of people (several hundred) to determine efficacy and to further evaluate its safety

5.0 **Describe the provisions made to break the code of a double-blind study in an emergency situation, and indicate who has the code (if applicable):**
We do not expect more than minimal risk in the study. Furthermore the patient and outcome assessor will be blinded in the study. The principal investigator will have access to the randomization code. Randomization will be documented in a separate chart available to the principal investigator.

6.0 **Provide justification for using placebo or no-treatment arm (if applicable):***(i.e. why/how is it OK to give a participant an inactive substance instead of a treatment)*
All patients will receive standard of care. Remote ischemic conditioning is a novel therapy. It has not been proven to effective in our study population. Hence to assess the true effect use of sham treatment is important.

7.0 **Describe the clinical criteria for withdrawing an individual participant from the study due to safety or toxicity concerns (if applicable):**
If patient develop discomfort to the study intervention (remote ischemic conditioning) which is more than usual an individual participants can withdraw from the study at any time. If there is change in patients clinical status due to factors not related to the study intervention (other post stroke complications) which require critical care the study intervention will be stopped. If the patient or family wishes to stop the study intervention to

change in the personal wishes or situation they can stop the therapy any time.

8.0 * **Expected Length/Duration of Clinical Trial***(in months)***:**
15

ID: Pro00117448 **Pro00117448**
Status: Approved 2.17 Data Safety and Monitoring for Clinical Trials

2.17 Data Safety and Monitoring for Clinical Trials

1.0 * **Check one that most accurately reflects the plan for data safety and monitoring for this study:**
The study will be monitored only by the study investigators.

2.0 * **Describe data monitoring procedures while research is going on. Include details of planned interim analysis, Data Safety Monitoring Board, or other monitoring systems:**
The principal investigator and co investigators will be responsible for the data monitoring. There is no planned interim analysis.

3.0 * **Summarize any pre-specified criteria for stopping or changing the study protocol due to safety concerns:**
We do not anticipate any adverse event related to the study intervention which will lead to stopping of trial.

ID: Pro00117448 **Pro00117448**
Status: Approved 2.19 Investigational Drugs, Devices, Biologics, Vaccines or Natural Health Products

2.19 Investigational Drugs, Devices, Biologics, Vaccines or Natural Health Products

1.0 **List all the investigational drugs, biologics, vaccine, natural health products, or devices used in the study. Enter the Health Canada No Objection Letter (NOL) control number and date of approval if available for the initial application and subsequent NOLs for amendments. Upload the NOL letter in the Documentation Section of your application.**

Name	Manufacturer	Type	Health Canada Approval Status	NOL Control Number	Date

There are no items to display

ID: Pro00117448 **Pro00117448** 3.1 Risk Assessment
Status: Approved

3.1 Risk Assessment

1.0 * **Provide your assessment of the risks that may be associated with this research:**
Minimal Risk - research in which the probability and magnitude of possible harms implied by participation is no greater than those encountered by

participants in those aspects of their everyday life that relate to the research (TCPS2)

2.0 * **Select all that might apply:**

Description of Possible Physical Risks and Discomforts

No	Participants might feel physical fatigue, e.g. sleep deprivation
No	Participants might feel physical stress, e.g. cardiovascular stress tests
Possibly	Participants might sustain injury, infection, and intervention side-effects or complications
No	The physical risks will be greater than those encountered by the participants in everyday life

Possible Psychological, Emotional, Social and Other Risks and Discomforts

No	Participants might feel psychologically or emotionally stressed, demeaned, embarrassed, worried, anxious, scared or distressed, e.g. description of painful or traumatic events
No	Participants might feel psychological or mental fatigue, e.g intense concentration required
No	Participants might experience cultural or social risk, e.g. loss of privacy or status or damage to reputation
No	Participants might be exposed to economic or legal risk, for instance non-anonymized workplace surveys
No	The risks will be greater than those encountered by the participants in everyday life

3.0 * **Provide details of all the risks and discomforts associated with the research for which you indicated YES or POSSIBLY above.**

All adverse events will be documents.
The remote ischemic conditioning intervention involves use of blood pressure cuff applied in the arm over the skin. The pressure is applied for 5 minutes followed by 5 minutes relaxation. This cycle is repeated for 5 times.
1. Rarely it may lead to discomfort due to the pressure sensation. Most people are able to tolerate the pressure sensation. If the person is not able to tolerate the intervention it can be stopped at any point. The sense of discomfort is transient and person returns to a state of baseline in few minutes time. In a research study, approximately 3.5% of people stopped the remote ischemic conditioning treatment due to discomfort.
2. Furthermore the pressure sensation may cause some transient superficial redness noted in upper arm skin. This change is transient as well. Which returns to baseline in few minutes time. In a research study, approximately 5.2% of people developed temporary redness in the arm where the pressure was applied.

4.0 * **Describe how you will manage and minimize risks and discomforts, as well as mitigate harm:**

1. To increase the comfort during the intervention we will be using an arm soft sleeve to be worn at the time of pressure application.
2. We will exclude patients with dermatological conditions precluding us from application of the pressure.
3. Patients with pre-existing pain concerns in upper arm will be exlcuded
4. To mitigate the risk of COVID 19 transmission the study investigator will use AHS guidelines during patient interaction.

5.0 **Is there a possibility that your research procedures will lead to unexpected findings, adverse reactions, or similar results that may require follow-up** *(i.e. individuals disclose that they are upset or distressed during an interview/questionnaire, unanticipated findings on MRI, etc.)***?**

○ Yes ● **No**

6.0 **If you are using any tests in this study diagnostically, indicate the member(s) of the study team who will administer the measures/instruments:**

Test Name	Test Administrator	Organization	Administrator's Qualification

There are no items to display

7.0 **If any research related procedures/tests could be interpreted diagnostically, will these be reported back to the participants and if so, how and by whom?**

ID: Pro00117448 **Pro00117448** 3.2 Benefits Analysis
Status: Approved

3.2 Benefits Analysis

1.0 * **Describe any potential benefits of the proposed research to the participants. If there are no benefits, state this explicitly:**
We anticipate no immediate benefit for the participants.

2.0 * **Describe the scientific and/or scholarly benefits of the proposed research:**
The study will help us understand
1. feasibility of remote ischemic conditioning device use in patients with acute ischemic stroke and small vessel disease
2. safety of remote ischemic conditioning device use in patients with acute ischemic stroke and small vessel disease

3.0 **If this research involves risk to participants explain how the benefits outweigh the risks.**
Remote ischemic conditioning has potential to reduce small vessel disease burden thus the study will allow us to plan phase III study. This will help in the advancement of science. The risk is minimal

ID: Pro00117448 **Pro00117448** 4.1 Participant Information
Status: Approved

4.1 Participant Information

1.0 * **Will you be recruiting human participants** *(i.e. enrolling people into the study, sending people online surveys to complete)***?**

● **Yes** ○ No

1.1 Will participants be recruited or their data be collected from

Alberta Health Services or Covenant Health or data custodian as defined in the Alberta Health Information Act?

● **Yes** ○ No

1.2 Would you like to include information about this study on the Be The Cure searchable database?

○ Yes ● **No**

4.2 Additional Participant Information

1.0 Describe the participants that will be included in this study. Outline ALL participants *(i.e. if you are enrolling healthy controls as well):*

All adult patients with ischemic stroke admitted to the University of Alberta Hospital stroke ward will be screened.

2.0 * Describe and justify the inclusion criteria for participants *(e.g. age range, health status, gender, etc.):*

1. Adult patients with ischemic stroke (anterior and posterior circulation involvement) with or without neurological deficit within 7 days of symptom onset
2. CT head or MRI Brain evidence of infarct
3. CT head or MRI Brain evidence of moderate or severe small vessel disease. We will assess atrophy, leukoaraiosis and old vascular lesions.
4. Premorbid functional disability assessed by mRS <2
5. National institute of Health Stroke scale <15 at the time of enrollment

3.0 Describe and justify the exclusion criteria for participants:

1. Patient is part of other clinical trial delivering intervention
2. Injury to the upper arm or any other musculoskeletal disability/pain precluding from tolerating RIC therapy
3. Treatment of ongoing malignancy with expected survival < 6 months
4. Presence of hypertensive urgency and emergency
5. Presence of hemodynamic instability
6. Presence of ongoing systemic infection with antibiotic therapy
7. Pregnant and lactating women
8. History of dermatological conditions affecting application of tissue perfusion sensor and remote ischemic conditioning pressure cuff

4.0 Participants

4.1 How many participants do you hope to recruit *(including controls, if applicable?)*
51
4.2 Of these, how many are controls, if applicable?
17
4.3 If this is a multi-site study, how many participants do you anticipate will be enrolled in the entire study?

5.0 Justification for sample size:
We will enroll a total of 51 (17 in Sham Control and 34 in the Intervention arm) patients over 9 months period.

ID: Pro00117448 **Pro00117448**
Status: Approved

4.3 Recruitment of Participants (Health)

1.0 Recruitment

* **1.1 How you will identify potential participants? Please be specific.** *(i.e. Will you be screening clinical lists, accessing electronic health records (e-clinician), asking staff from a particular area to let you know when a patient meets criteria, will you be sitting in the emergency department waiting room, etc?)*
The attending teams would be alerted about the study at the beginning of the week. They would approach the patient to seek permission if they would like to hear more about the study. If the patient or family members (when the patient is incapacitated) agree to hear about the study. The attending team would inform the study team member to approach the patient to inform about the study and answer questions.

1.2 If you are using patient/clinical records to identify potential participants for research purposes, will someone from the data custodian/clinical care team seek prior consent of the participant to allow the researcher to look at their records?
● **Yes** ○ No

1.3 Once you have identified a list of potentially eligible participants, indicate how the potential participants' names will be passed on to the researchers AND how will the potential participants be approached about the research.
The stroke ward team will inform the research coordinator of potential patients or caregivers who have agreed to speak to the team verbally or via connectcare message. The potential participants or caregivers will be approached in the stroke ward at a time convenient to them.

1.4 Outline any other means by which participants could be identified*(e.g. response to advertising such as flyers, posters, ads in newspapers, websites, email, list serves, physicial or community organization referrals):*
Only through the stroke ward team

2.0 Pre-Existing Relationships

2.1 Will potential participants be recruited through pre-existing relationships with researchers *(e.g. Will an instructor recruit students from his classes, or a physician recruit patients from her practice? Other examples may be employees, acquaintances, own children or family members, etc)***?**
○ Yes ● **No**

3.0 **Will your study involve any of the following** *(select all that apply)***?**
None of the above

4.5 Informed Consent Determination

1.0 **Describe who will provide informed consent for this study***(i.e. the participant, parent of child participant, substitute decision maker, no one will give consent – requesting a waiver)*
The informed consent will be provided either by the participant or the substitute decision maker. Once the Participant regains capacity they will then sign a regained capacity consent form.

1.1 Waiver of Consent Requested
If you are asking for a waiver of participant consent, please justify the waiver or alteration and explain how the study meets all of the criteria for the waiver. Refer to Article 3.7 of TCPS2 **and provide justification for requesting a Waiver of Consent for ALL criteria (a-e)**
NA

1.2 Waiver of Consent in Individual Medical Emergency
If you are asking for a waiver or alteration of participant consent in individual medical emergencies, please justify the waiver or alteration and explain how the study meets ALL of the criteria outlined in Article 3.8 of TCPS2 (a-f)**.**
NA

2.0 **How will consent be obtained/documented? Select all that apply**
Signed consent form

If you are not using a signed consent form, explain how the study information will be provided to the participant and how consent will be obtained/documented. Provide details for EACH of the options selected above:

3.0 **Will every participant have the capacity to give fully informed consent on his/her own behalf?**
○ Yes ● **No**

3.1 Explain why participants lack capacity to give informed consent *(e.g. age, mental or physical condition, etc.).*
Due the ongoing neurological deficit (including but not limited to Aphasia) not all participants will have the capacity to give consent.

3.2 Will participants who lack capacity to give full informed consent be asked to give assent?
○ Yes ● **No**

3.3 In cases where participants (re)gain capacity to give informed consent during the study, how will they be asked to provide consent on their own behalf?
If the study team member observes that participant has regained capacity, they will confirm with the attending team if they agree with that assessment. Once the attending team also agrees we will approach the participant with the regained capacity consent form.

4.0 **What assistance will be provided to participants or those consenting on their behalf, who may require additional assistance?** *(e.g. non-English speakers, visually impaired, etc.)*
The study team will make every effort so that the participants and the substitute decision makers are able to provide an informed consent. For visually impaired we will read the informed consent so that they can make better decision. We will request if a family member is present either in person or on phone who can understand English.

5.0 * **If at any time a PARTICIPANT wishes to withdraw from the study or from certain parts of the study, describe when and how this can be done.**
Any participants can withdraw from the study at any time. They will have to inform the stroke ward team or research team about the same. As this is a clinical trial we will continue to store data that has been collected so far. If the family or participants are willing to answer a follow up telephone call in 3 months we would call. If they are not willing we will consider them withdrawn. The statistical analysis is intention to treat hence all enrolled patients will be included for analysis.

6.0 **Describe the circumstances and limitations of DATA withdrawal from the study, including the last point at which participant DATA can be withdrawn** *(i.e. 2 weeks after transcription of interview notes)*
Data collection can be stopped immediately at the time of withdrawal. A withdrawal consent form will be presented to the participant. Further proceedings of the study will be in keeping with regained capacity consent form and withdrawal consent forms.

7.0 **Will this study involve any group(s) where non-participants are present? For example, classroom research might involve groups which include participants and non-participants.**
○ Yes ● **No**

ID: Pro00117448 **Pro00117448** 5.1 Data Collection
Status: Approved

5.1 Data Collection

1.0 * **Will the researcher or study team be able to identify any of the participants at <u>any stage</u> of the study?**
● **Yes** ○ No

2.0 **Primary/raw data collected will be** *(check all that apply)*:
Directly identifying information - the information identifies a specific individual through direct identifiers (e.g. name, social insurance number, personal health number, etc.)

Indirectly identifying information - the information can reasonably be expected to identify an individual through a combination of indirect identifers (eg date of birth, place of residence, photo or unique personal characteristics, etc)

All personal identifying information removed (anonymized)

3.0 **If this study involves secondary use of data, list all original sources:**
No secondary use of data

4.0 **In research where total anonymity and confidentiality is sought but cannot be guaranteed** *(eg. where participants talk in a group)* **how will confidentiality be achieved?**
All data collected will be anonymized

5.2 Data Identifiers

1.0 * **Personal Identifiers:** will you be collecting - at any time during the study, including recruitment - any of the following *(check all that apply)*:
Surname and First Name
Telephone Number
Age at time of data collection

2.0 **Will you be collecting - at any time of the study, including recruitment of participants - any of the following** *(check all that apply)*:
Hospital Discharge Date
Other Date (eg Date of Service)

3.0 * **If you are collecting any of the above, provide a comprehensive rationale to explain why it is necessary to collect this information:**
1. Age at the time of data collection: Age is a determinant of the study intervention
2. Name and Telephone number: Patient will have a telephone follow up at 3 months. The name will be used to address the patient or to speak with caregiver.
3. Hospital discharge date will be documented to calculate how many days patients received study intervention.
4. Other date: date of admission, date of start of study intervention

4.0 **If identifying information will be removed at some point, when and how will this be done?**
At the time of data analysis direct identifying information will be removed. A separate excel master sheet will be created. All patients will be allotted a study number.

5.0 * **Specify what identifiable information will be RETAINED once data collection is complete, and explain why retention is necessary. Include the retention of master lists that link participant identifiers with de-identified data:**
Age at the time of data collection, hospital discharge date and date of admission will be retained. Age is important outcome determinant in recovery and occurrence of stroke. Hospital discharge date and admission date will be documented on the case record form and will be used to

calculate the duration of stay in the hospital. They will be stored on the case record form and excluded from the excel sheet.

6.0 **If applicable, describe your plans to link the data in this study with data associated with other studies (e.g within a data repository) or with data belonging to another organization:**
No plan to link the study to other data base

ID: Pro00117448 **Pro00117448** 5.3 Data Confidentiality and Privacy
Status: Approved

5.3 Data Confidentiality and Privacy

1.0 * **How will confidentiality of the data be maintained? Describe how the identity of participants will be protected both during and after research.**
Each patient will be assigned a study number. The study case record form will be maintained in locked cabinet with access only to two study team members (MPK, RKN). The case record form will be maintained after the research is completed according to the Alberta Health services and University of Alberta rules and regulation for research data.

2.0 **How will the principal investigator ensure that all study personnel are aware of their responsibilities concerning participants' privacy and the confidentiality of their information?**
All study team members gave completed a Good Clinical Practice (GCP) and Health Canada Division 5 training. A study log will be maintained about the uptodate certificates.

3.0 **External Data Access**

* **3.1 Will identifiable data be transferred or made available to persons or agencies outside the research team?**
○ Yes ● No

ID: Pro00117448 **Pro00117448** 5.4 Data Storage, Retention, and Disposal
Status: Approved

5.4 Data Storage, Retention, and Disposal

1.0 * **Describe how research data will be stored, e.g. digital files, hard copies, audio recordings, other. Specify the physical location and how it will be secured to protect confidentiality and privacy.** *(For example, study documents must be kept in a locked filing cabinet and computer files are encrypted, etc. Write N/A if not applicable to your research)*
The data will be stored in physical form as case record form. Data obtained during the study intervention and sham is anonymized according to the patient study number. It will be in digital format. The anonymized data from the case record form and study intervention data will then be transferred to excel sheet.

1. *Check that the named investigators on your application match the people named on the RES account. RES numbers associated with anyone named as PI or Co-I on an ethics application will show up in the drop down box in Section 1.3 or in 6.0 of the Change Funding Activity. Please note that unless someone is named on the ethics application in either the PI or Co-I fields, their RES number(s) will NOT display in the drop down box of that application.*
2. *Check that the RES number you are trying to add has been activated by RSO (check unit name with RSO) and that 24 hours have elapsed since it was activated to allow time for system updates.*

If neither of the above items are the source of the issue, please contact reoffice@ualberta.ca.

Enter your Peoplesoft Project ID (aka RES#) to link this ethics application to the project record in PeopleSoft.

PeopleSoft Project ID:
RES0055934

Other Relevant Information:
